# Operationalizing Implementation Science in Nutrition: The Implementation Science Initiative in Kenya and Uganda

**DOI:** 10.1093/cdn/nzab146

**Published:** 2021-11-29

**Authors:** Isabelle Michaud-Létourneau, Marion Gayard, Brian Njoroge, Caroline N Agabiirwe, Ahmed K Luwangula, Laura McGough, Alice Mwangi, Gretel Pelto, Alison Tumilowicz, David L Pelletier

**Affiliations:** The Society for Implementation Science in Nutrition, NY, USA; Department of Social and Preventive Medicine, School of Public Health, University of Montréal, Montréal, Québec, Canada; The Society for Implementation Science in Nutrition, NY, USA; FHI Partners, Nairobi, Kenya; University Research Co., LLC, Kampala, Uganda; University Research Co., LLC, Kampala, Uganda; University Research Co., LLC, Kampala, Uganda; FHI Partners, Nairobi, Kenya; The Society for Implementation Science in Nutrition, NY, USA; Division of Nutritional Sciences, College of Human Ecology, Cornell University, Ithaca, NY, USA; The Bill and Melinda Gates Foundation, Seattle, WA, USA; The Society for Implementation Science in Nutrition, NY, USA; Division of Nutritional Sciences, College of Human Ecology, Cornell University, Ithaca, NY, USA

**Keywords:** implementation science, operationalization, nutrition programs, developmental evaluation, systems, capacity-building, knowledge brokering

## Abstract

**Background:**

Implementation science (IS) has the potential to improve the implementation and impact of policies, programs, and interventions. Most of the training, guidance, and experience has focused on implementation research, which is only 1 part of the broader field of IS. In 2018, the Society for Implementation Science in Nutrition borrowed concepts from IS in health to develop a broader and more integrated conceptual framework, adapted to the particular case of nutrition and with language and concepts more familiar to the nutrition community: it is called the IS in Nutrition (ISN) framework.

**Objective:**

The purpose of this research was to generate knowledge concerning challenges and strategies in operationalizing the ISN framework in low- and middle-income country (LMIC) settings.

**Methods:**

The ISN framework was operationalized in partnership with country teams in Kenya and Uganda over a 3-y period as part of the Implementation Science Initiative. An action research methodology (developmental evaluation) was used to provide timely feedback to the country teams, facilitate adaptations and adjustments, and generate the data presented in this article concerning challenges and strategies.

**Results:**

Operationalization of the ISN framework proceeded by first articulating a set of guiding principles as touchstones for the country teams and further articulating 6 components of an IS system to facilitate development of work streams. Challenges and strategies in implementing these 6 components were then documented. The knowledge gained through this experience led to the development of an IS system operational model to assist the application of IS in other LMIC settings.

**Conclusions:**

Future investments in IS should prioritize a system- and capacity-building approach in order to realize its full potential and become institutionalized at country level. The operational model can guide others to improve the implementation of IS within a broad range of programs.

## Teaser

Implementation science is broader and more complex than implementation research and requires a wider range of strategies to build national capacity and fulfill its potential.

## Introduction

Implementation science (IS) is recognized to have great potential to assist in the translation of policies, programs, and interventions into population-level impact (1, 2). In recent decades there have been increases in funding, training, and publishing of implementation research (IR), notably in relation to the delivery of health services and in high-income countries (3–8). This has led to impressive conceptual, theoretical, and methodological advances, for instance, to enhance the selection and use of theory, models, and frameworks (9, 10); identify and measure implementation barriers and enablers (11–13); and select, design, implement, and report strategies to overcome specific barriers (14–17), in addition to the development of frameworks for the design of overall IS systems themselves (18–20). Although the development of the field in these high-income settings is welcome, progress has been slower in low- and middle-income countries (LMICs) (21–25).

The Society for Implementation Science in Nutrition (SISN) was formed in 2016 to promote and support the application of IS in nutrition (ISN), with a particular focus on undernutrition in LMICs (26). This was a response to the increasing recognition of the important contributions of nutrition to the global burden of disease and human capital (27, 28), the ascendancy of nutrition on global and national policy agendas (29, 30), and the relative neglect of implementation issues in nutrition research agendas (31–34).

Recognizing that most nutrition researchers and practitioners have had relatively little exposure to IS, SISN borrowed concepts from IS in health to develop a guiding conceptual framework, the ISN framework, adapted to the particular case of nutrition and the language and concepts more familiar to the nutrition community (35). SISN then obtained funding for a 3-y initiative to generate knowledge concerning challenges and strategies in operationalizing the ISN framework in LMIC settings, in order to inform future efforts to build national capacity for IS in such settings. The present article describes the experiences gained from this initiative and the operational model that emerged. The primary audiences for this article are individuals and organizations interested in building national capacities for IS.

An overview of the ISN framework guiding the initiative is presented next, with more details available in the original article (35). This is followed by a description of the methodology and results from the 2-country initiative, as well as the operational model emerging from the experience. Overall reflections and conclusions are provided in the final section.

### A conceptual framework for IS in nutrition

**FIGURE 1:**
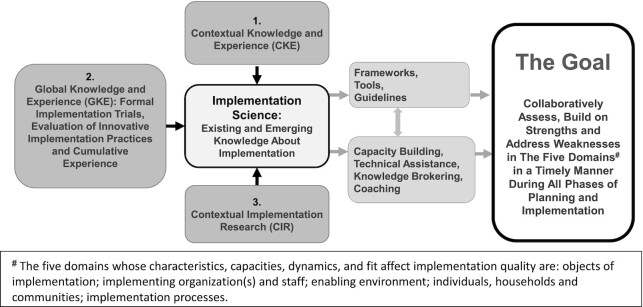
The Society for Implementation Science in Nutrition's Integrated Framework for Implementation Science in Nutrition. Adapted from (35) (copyright *Current Developments in Nutrition*).

The ISN framework interprets IS as the science of implementation, that is, the existing and emergent body of knowledge about ways to strengthen the implementation of policies, programs, interventions, and innovations (**Figure 1**). It explicitly recognizes that 3 forms of knowledge contribute to the science of implementation: contextual knowledge and experience (CKE), as possessed by practitioners or actors in the system; global knowledge and experience (GKE), as found in scientific and gray literature; and contextual implementation research (CIR), representing new empirical inquiries in the local or national context. These distinctions are fundamental for several reasons: they acknowledge the vital and equal roles of local actors as knowledge producers, providers, and translators, not simply as implementers; they acknowledge that many implementation problems can be addressed with existing implementation knowledge and experience from the same setting or other global settings; and they clarify the conceptual difference between IS (a broad body of knowledge) and IR (new empirical inquiries undertaken selectively when the other forms of knowledge are not sufficient). This broader and more integrated view of implementation knowledge is transformative because it enhances the ability of IS to be relevant to implementers, governments, and donors, but using methods that are practical, timely, and appropriately rigorous for the decisions at-hand (36, 37).

The ISN framework highlights that the uptake and utilization of implementation knowledge requires that it be converted into practical tools, frameworks, and guidelines; and various types of purveyors (mentors, coaches, etc.) play critical roles in identifying, creating, or adapting these tools, as well as assisting in their uptake and correct utilization (38). These elements of the framework also are fundamental because, along with the reference to “collaborative” in the goal statement, they underscore the need for explicit strategies (and actors) to ensure knowledge translation and utilization. Finally, as noted in the reference to “all five domains” in the goal statement, the framework takes a broad view of the range of factors that can affect implementation, building upon the Consolidated Framework for Implementation Research (11) and adapting it to the language and contexts of nutrition in LMICs (35). The 5 domains are as follows: the objects of implementation (particular interventions or policies); implementing organizations and staff [e.g., nongovernmental organizations (NGOs) or ministries]; the enabling environment (policies and governance capacities); individuals, households, and communities (as the end users); and implementation processes (e.g., quality of planning, stakeholder engagement, training). Further details on these 5 domains are available in the original framework article (35).

Taken together, these key elements of the ISN framework acknowledge that *1*) implementation takes place within multilevel systems and domains, *2*) IS may be needed to understand and address challenges in any of these domains, and *3*) IS itself can be viewed as a system (with distinct components to mobilize existing knowledge, to generate new knowledge, and to ensure its appropriate uptake and utilization by decision makers).

This is in contrast to discrete and time-bound IR projects to address specific bottlenecks in a particular program, which is the more common practice in LMICs (22).

### The Implementation Science Initiative

The Implementation Science Initiative (ISI) was conducted in 2018–2021 in Kenya and Uganda to gain experience in operationalizing, or putting into practice, the concepts embodied in the ISN framework. It used a focal intervention, iron and folic acid supplementation (IFAS) for pregnant women, as an entry point to learn how to build national capacity for IS and enable SISN to develop tools and approaches that could be applied elsewhere in the future. Specifically, the 4 main objectives were to:

1. strengthen implementation of IFAS programs;
2. strengthen interaction and knowledge exchange among key actors (policy, program, and research);
3. strengthen capacity for applying IS at the country level; and
4. increase knowledge about how to apply IS in LMIC settings.

This article presents the results pertaining to objective 4.

The initiative was carried out as part of a partnership with the International Initiative for Impact Evaluation (3ie) thanks to financial support from the Bill & Melinda Gates Foundation.

In each country, a team of program implementers and researchers was formed to collaborate with SISN in operationalizing the ISN framework in 1 of their ongoing health programs. In Kenya the implementing partner was FHI Partners and the research partner was the Kenya Medical Research Institute. The focal program was Nutrition and Health Plus Program, which was operating in 5 counties. In Uganda the implementing partner was University Research Co., LLC (URC) and the research partners were from the Makerere University School of Public Health. The focal program was the Regional Health Integration to Enhance Services in East Central Region (RHITES-EC) program working in 11 districts of this region. In both cases these NGO-assisted focal programs were operating through Ministry of Health structures at clinic and community levels. IFAS for pregnant women was nominally part of the maternal and child health service package but the state of its implementation was not well documented. The Kenya National Micronutrient Survey 2011 indicated that the national prevalence of *1*) anemia in pregnant women was 41.6%; *2*) iron deficiency among pregnant women was 36.1%; and *3*) iron deficiency anemia was 26%. The national prevalence of anemia among pregnant women was 34% in Uganda. IFAS is part of the national nutrition policy or strategy in both countries.

## Methods

This initiative was designed by SISN to learn and document how to operationalize the ISN framework by working in collaboration with 2 country teams, fully anticipating there would be a range of complications and challenges but not fully knowing in advance what they would be. For that reason, developmental evaluation was chosen as the operationalization framework as well as the research methodology. Developmental evaluation is a systematic and rigorous approach that supports the development and implementation of an innovation in a complex and dynamic context by collecting various types of data and providing timely feedback to implementers on how to make adaptations (39). In this case, the “innovation” was the ISN framework.

In this initiative, developmental evaluation was used for 2 purposes: *1*) as a facilitation and capacity-building methodology to provide timely feedback to the country IS teams and facilitate adaptations and adjustments; and *2*) as a research methodology to document and disseminate the experiences of operationalizing the ISN framework in the 2 countries. Developmental evaluation requires close engagement of a researcher with the main actors engaged in implementing the innovation. Over a 30-mo period, the SISN senior technical lead (IM-L) interacted with the country teams, primarily remotely, and played multiple roles. These included asking evaluative questions and gathering information to provide feedback; facilitating reflection and supporting decisions and adaptations in real time; sharing experiences across the 2 countries; creating access to GKE related to IFAS programs; and providing technical assistance on how to apply the ISN framework. In addition, she was gathering the data on the challenges and strategies in operationalizing the ISN framework. Whereas SISN led the reflexive process through developmental evaluation and provided various types of guidance, the country teams led the efforts to operationalize the ISN framework and make adjustments within their respective country and selected program.  ** Table 1** presents the data collected and **Table 2** presents products generated from using the developmental evaluation approach.

**FIGURE 3:**
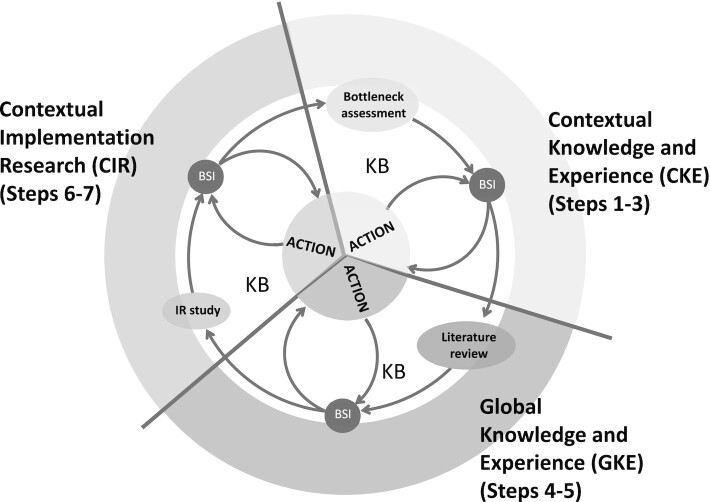
An operational model of the Implementation Science System. BSI, bottleneck and solution inventory; IR, implementation research; KB, knowledge brokering.

**TABLE 1:** Data collected as part of developmental evaluation ^1^

Methods	Purpose	Activities
Desk review	Provide background information on the programs implemented by the 2 implementing agencies and the context	– Review of various documents such as program reports (from implementers); national documents on IFAS program; relevant national policies; articles on related topics in the countries
Regular calls	*1*) Document the experiences; *2*) provide updates on the work carried out in each country; *3*) reflect on challenges and try to develop strategies to address them; *4*) discuss comments on products; *5*) adapt to the evolving context and specific situations; *6*) consult with the broader team; *7*) share between the 2 teams; *8*) prepare for webinars or build capacity on a certain topic; *9*) carry out a particular process	– Numbers: >40 calls with Kenya team; >50 calls with Uganda team; 12 calls with both countries– Participants: primarily project coordinators; other key actors: nutrition advisor, (deputy) chief of party, researchers– Topics: follow-ups on components; implementation challenges and solutions; contextual factors; suggestions and potential actions; accomplishments; problems; etc
Participant observation/key informant meetings	*1*) Tailor the activities based on opportunities; *2*) document the experiences; *3*) intensify the work and coordinate with stakeholders in-country	– Three technical assistance visits in both countries, involving meetings with project coordinators, members of the implementing teams, members of the core teams, and other key stakeholders at national and subnational levels

^1^IFAS, iron and folic acid supplementation.

Ethical approval was granted from the Institutional Review Board committee of Cornell University for the overall research and from Kenya Medical Research Institute (KEMRI) in Kenya and The AIDS Support Organization (TASO) in Uganda for the research in those countries.

**TABLE 2:** Products generated as part of developmental evaluation^1^

Types of document	Purpose	Content
Guidance documents and tools	Provide practical guidance to country teams on various aspects of the initiative	*1*) Guidance notes on 6 components of ISI; theory of change; knowledge brokering; bottleneck assessment; reflective practice; inquiry approach;*2*) Reflexive exercise for the bottleneck inventory
Briefs or short documents	*1*) Present the initiative to several groups including the selected implementers and share key concepts in implementation science; *2*) build capacity by presenting less conventional methodologies relevant for ISI	*1*) Flyers on implementation research; Brief: The Implementation Science Initiative in Kenya and Uganda; *2*) Briefs: What is a focused ethnographic study?; What is effectiveness-implementation hybrid design?
Reports	*1*) Document the initiatives and articulate next steps by components or objectives; *2*) carry out a preliminary analysis on the operationalization of the initiative, using logic models; *3*) provide an analysis of 1 specific component that presents challenges and propose ways forward	*1*) Progress reports: inception workshop report, technical assistance trip reports; *2*) interim report on the operationalization of ISI; *3*) bottlenecks and solutions inventory report
Documents created from literature searches	*1*) Guide the proposal development by formulating a menu of options adapted to the contexts; *2*) build capacity on new methodologies and articulate considerations for the teams; *3*) develop a framework to help classify factors influencing program implementation and adherence to interventions	*1*) Review of a selected literature on the determinants of compliance with IFAS; considerations for research (Kenya: formulation of objectives; Uganda: methodology); *2*) undertaking a process evaluation (Uganda); using effectiveness-implementation hybrid design; *3*) using focused ethnographic study; *4*) exploration of frameworks to classify various factors related to IFAS
Living documents	Document challenges, responsive actions to respond to country needs, and processes related to the set-up of ISI and its operationalization	*1*) List of responsive feedback; *2*) table on operationalization of the components by the countries; *3*) SISN support over the initiative; chronologies of events

1 IFAS, iron and folic acid supplementation; ISI, Implementation Science Initiative; SISN, Society for Implementation Science in Nutrition.

## Results

### Operationalization of the ISN framework

**FIGURE 2:**
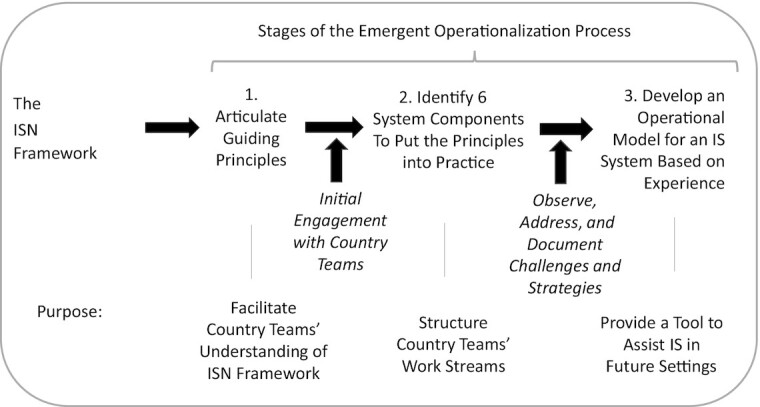
Emergent process for operationalizing the ISN framework. IS, implementation science; ISN, Implementation Science in Nutrition.

Throughout the initiative, a reflexive and adaptive process took place for the operationalization of the ISN framework. **Figure 2** shows the evolution of this process. The presentation of results and discussion in this article is organized in relation to the 3 stages shown in the figure.

#### Guiding principles

The development of guiding principles early in an initiative is one of the key practices in developmental evaluation, to provide touchstones for implementers. As stated in a development evaluation toolkit, guiding principles are useful “in a complex initiative where you're making your way through unmapped territory. You may not always have a clear sense of where you are or what comes next, but you'll be able to chart direction and progress if you have an actionable focus and meaningful guiding principles” (40).

Five guiding principles were articulated in this initiative, to facilitate the country teams’ understanding of the ISN framework:

1. Mobilize existing knowledge, frameworks, and tools to address some of the bottlenecks whenever possible;
2. When research is needed, use methods with the level of rigor, practicality, and timeliness appropriate to the decision context;
3. Collaboratively identify research topics based on priority implementation challenges and bottlenecks, recognizing that these may exist in any of the 5 domains;
4. Facilitate formal and informal interaction, knowledge exchange, and collaboration between researchers and program/policy actors in an ongoing manner in order to foster common understandings, effective working relationships, and appropriate interpretation and application of IS findings;
5. Conduct prospective documentation and reflection on the emergent processes in an ongoing manner in order to make strategic adjustments in the current project and generate the data needed to evaluate and disseminate broader lessons for global audiences.

#### Six components to put the principles into practice

As SISN engaged with the country teams, it became clear that more guidance was needed on how to apply the ISN framework and guiding principles. To that end, 6 components were articulated to provide greater specificity to the implementing agencies’ activities and work streams. Taken together, these 6 components represent the elements of an “IS system” designed to address implementation bottlenecks in a systematic, efficient, and practical manner and create a structure (the IS network) that could facilitate IS capacity building over time. The rationale and initial vision for each of those IS system components are described next, along with the challenges encountered in implementing them and the strategies and activities mobilized in an attempt to address them. **Table 3** details the latter. Note that BNA and BNI are presented separately in the table as those involved distinct challenges and strategies.

**TABLE 3:** Challenges and strategies implementing the 6 components of the IS System^1^

Component	Challenges in operationalizing the component	Strategies and actions
Knowledge brokering	Lack of familiarity with knowledge brokering, how it relates to the 3 forms of knowledge, and how it is needed to address bottlenecks and apply IS	– Development of a knowledge brokering strategy and guidance– Designate the project coordinator as the leading knowledge broker– Assistance to support the diverse applications of knowledge brokering
National core team	– Competing priorities and limited engagement of actors – Members primarily engaged to develop and carry out the IR, which can divert attention from the other components– Lack of understanding of the distinction between IS and IR– Frequent turnover of the team members	– Create terms of reference for the core team– Create subgroups around the interests of the members to maximize participation, consider their incentives, and build on strengths– Plan a core team meeting when SISN made country visits– Follow up with individual members on their areas of interest– Share progress on the work (e.g., milestones regarding the IR)– Continuous efforts to build understanding of IS and maintain trust among members
BNA	– Assumption that the purpose of the BNA was to identify IR topics– No systematic tool or procedure for the BNA– No specific exercise for the prioritization of bottlenecks – Existing BNA guides or tools may not cover the user system (i.e., client, household, and community)	– Collect preliminary data to draw an initial picture of the situation and engage with stakeholders– Organize a BNA workshop through a participatory approach using an existing tool (Program Assessment Guide)– Do a pair-wise ranking to prioritize the bottlenecks– Anticipate the need for a literature review to identify potential bottlenecks in the user system and seek supplemental funding for IR to explore such bottlenecks in the local context
BSI	– No previous experience with a BSI– Technological and conceptual challenges and delays when trying to use an existing platform for the BSI– Potential sensitivity of referring only to bottlenecks– No guidance on how to use existing knowledge to address bottlenecks before moving into IR studies– Inflexible adherence to the original work plan	– Develop a template to classify the bottlenecks– Create a guidance note and an exercise to create a process for the BSI (Before Action Review–After Action Review)– Pilot test the exercise with the core team members– Change the name from Bottleneck Inventory to BSI to minimize sensitivities and emphasize the focus on solutions– Exercise caution in how the bottlenecks are framed
IR	– Assumption that the purpose of the BNA was to identify IR topics– Uncertainty in how to go from the bottlenecks to IR questions– Excessive time and effort to create IR and get Institutional Review Board approvals, which detracted from the other IS components– Turnover in core team members and challenges in designating roles for management as opposed to research	– Establish a collaborative process for the development of the protocol– Provide capacity-building opportunities on different methodologies (e.g., focused ethnographic study; effectiveness-implementation hybrid design)– Provide technical assistance at each stage of the research– Field visit to better understand the context and interventions– Organize a process to share among countries and interact with experts on certain methodologies
IS network	– Lack of clarity on purpose of the IS network– No systematic process for the creation of an IS network– Lack of clarity on who could engage in a network and on who would assume the leadership	– Develop terms of reference for the network– Consider the readiness of a country to host an IS network– Work with some national core team members to examine potential avenues to create the IS network– Engage with an existing network (SUN Academic network)– Plan a series of webinars on IS to build the capacity of stakeholders– Find a champion to lead the IS network, a strategic home, and a rotating home
Ongoing documentation of experiences	Project coordinators in-country had a large workload with the implementation of ISI in-country and limited time to take charge of documenting their experience	– Carry out multiple reflective practice calls between the SISN senior technical lead and the project coordinators to document country experiences and support the development of their innovation– Use all the documents for the other documents to track progress and generate insights

^1^BNA, bottleneck assessment; BSI, bottleneck and solution inventory; IR, implementation research; IS, implementation science; ISI, Implementation Science Initiative; SISN, Society for Implementation Science in Nutrition.

*1*) Knowledge brokering. Knowledge brokers are people who are specifically tasked with facilitating the access, interpretation, adaptation, and utilization of knowledge (38, 41, 42). This concept was adapted in the initiative to include all 3 forms of knowledge in the ISN framework described earlier (CKE, GKE, and CIR), all of which can play a role in addressing implementation bottlenecks. In this initiative country-based knowledge brokers played key roles in liaising with varied stakeholders in order to implement the various components of the IS system. Senior staff from FHI and URC were supported (part-time) by the project's grant funds, designated as overall country coordinators, and played the role of knowledge brokers. They were supported by and worked closely with the SISN senior technical lead in all aspects of the work.

The concept of knowledge brokering resonated with the country teams because many members had played similar roles in previous projects, but without calling it as such. This is common because, although the concept emerged in the early 2000s within health care (43) and the literature is flourishing (38, 41, 42), people are rarely called knowledge brokers. SISN mobilized a number of strategies to gradually build awareness, capacity, and interest in playing the various knowledge brokering roles (Table 3). Over time the knowledge brokering lens became useful for the country teams who gained valuable experience in formalizing this set of practices. Given the central importance of knowledge brokering for the success of any IS initiative this is examined in greater detail in a later section.

*2*) Bottleneck assessment and bottleneck inventory. An early and foundational step in the IS system operational model was for the country teams to identify and document implementation bottlenecks (a bottleneck assessment), and then create and use a tool to track and maintain progress in addressing them [a bottleneck inventory, which later became a bottleneck and solution inventory (BSI) to keep it positive and include solutions]. This was a daunting task, given the multiple domains in which bottlenecks can occur (from the enabling environment, through the delivery system, and in the user system). Although the country teams were cognizant of some bottlenecks in their programs, they did not have experience with a more systematic process from the perspective of the entire implementation system. The SISN team introduced 2 tools from the literature and worked with the country teams to adapt them to their context. These tools are the Program Assessment Guide (PAG) (44), which was used for the bottleneck assessment, and the Before and After Action Review (45), which was used for the BSI.

The PAG is a structured participatory process for a 3- to 5-d workshop for rigorously eliciting and systematizing CKE to strengthen the design and delivery of interventions on a large scale (44). The PAG workshop participants are chosen from all levels and components of the delivery system (e.g., service delivery, counseling, and supply chain), to draw upon their contextual knowledge about their portion of the system. It also helps build a common understanding of the system as a whole and build buy-in for improving its performance. This tool gave the country teams a concrete way to conduct the bottleneck assessment and both countries designed and implemented a successful PAG workshop. It is important to note that PAG workshops do not include mothers, caregivers, and others in the user system at community level, so other methods (notably literature reviews and IR) are needed to identify bottlenecks in that domain. Supplementary grant funds were provided to both teams to conduct a focused ethnographic study (46) for this purpose, focusing on early disclosure of pregnancy, antenatal care (ANC) attendance, IFAS adherence, and male involvement in ANC, although these studies were not conducted owing to COVID-19.

*3*) IR. Whereas guiding principle #1 emphasizes the use of existing GKE and CKE to address some of the bottlenecks whenever possible, guiding principles #2 and #3 recognize that IR often is needed as well. Within the ISN framework, IR includes a wide range of practical and timely assessments and inquiries (e.g., formative research, rapid assessments, stakeholder analysis) to better understand bottlenecks and potential solutions in any of the 5 domains. This is distinct from more formal approaches that require more time, resources, and technical capacities (e.g., effectiveness trials).

A major challenge emerged in the initiative because proposals for IR were developed too early in the process. This absorbed a large amount of attention, time, and resources and detracted from the ability to address some of the bottlenecks immediately and to work on other components. Several factors contributed to this, including the way in which the project budgets were structured at the outset (which presumed that such IR studies would be conducted), the tacit assumption that such studies are the core component of IS, and the professional incentives of researchers on the country teams.

*4*) National core team. In line with guiding principle #4, both countries made efforts to form an ad hoc structure (a national core team) in which implementers and researchers could interact with policy makers to share emerging experiences, mobilize knowledge and perspectives from key stakeholders, and access the authority to address the identified bottlenecks. In Kenya this core team included actors from the Ministry of Health, KEMRI, and Nutrition International; in Uganda it included actors from the Ministry of Health, Office of the Prime Minister, and district offices. Several factors prevented these core teams from meeting and functioning as intended: competing time demands, the focus on the formal IR studies when they did meet, and the onset of COVD-19 which prevented the implementing teams from forming concrete bottleneck solutions for consideration by policy makers. Two strategies that provided a partial workaround were to schedule core team meetings during country visits by the SISN senior technical lead and having the country knowledge broker engage with individual members outside of formal meetings.

*5*) IS Network. Whereas the national core teams were envisioned as a mechanism to facilitate interaction among actors most relevant to the IFAS intervention, the IS network was envisioned as a strategy to build interest in IS among a broader range of health and nutrition stakeholders at the national level. This component was articulated in an effort to lay the groundwork for future capacity building, upscaling, and sustainability of IS in the country by sharing experiences and lessons emerging from the ISI, eliciting experiences from other members of the network, and providing a platform for discussing future IS initiatives. It was expected that the country knowledge brokers would play a key role in catalyzing these networks.

This component of the IS system was given lower priority during the first 2 y of the initiative because of the time devoted to the other components (notably the development of IR proposals) and the lack of clarity concerning the purpose of the IS network and how to go about creating it. With the onset of COVID-19 and interruption of the planned IR in Kenya, the knowledge broker in that country was able to devote more time to the IS network toward the end of the initiative. By early 2021, when external funding was terminated, 2 options were being explored. One was to convene interested organizations on a regular basis and rotate the responsibilities for convening across these organizations to broaden ownership and buy-in and prevent rivalries. A second was to stimulate interest in IS on the part of members of the Scaling Up Nutrition (SUN) movement Academic and Research Network.

*6*) Ongoing documentation of experiences. This sixth component was included in order to facilitate real-time reflection and adjustments by and with the country teams and to generate the data needed to evaluate and disseminate broader lessons for global audiences. Developmental evaluation was the methodology for both of these purposes. Both of these purposes may be of interest in future IS initiatives, although some may wish to focus primarily on the first of these in order to meet the immediate needs of implementers.

#### Development of an IS system operational model

Based on the challenges in operationalizing the ISN framework and the strategies used to address them, we articulated an operational model of how an IS system can work and some guidance that can assist the process. This model is described here and referred to as an IS system operational model. The corresponding guidance is provided in an accompanying guide (47).

The IS system operational model involves 7 steps, presented as a cycle (**Figure 3**) to emphasize that in most cases they are carried out in a sequential and iterative manner. Decisions and actions are at the center of the cycle because they are central to the goal of addressing the identified bottlenecks. The outer circle depicts the 3 different forms of knowledge to be mobilized and translated into action. **Table 4** presents an overview of the 7 steps.

**TABLE 4:** Overview of the Implementation Science System operational model^1^

Step	Description
1. BNA	– Assessment done in a program to identify bottlenecks at various levels in the systems and potential solutions, building on contextual knowledge and experience– Prioritization done at the end of the BNA to reach agreement on next steps
2. BSI	– Living document updated over time that gathers all the bottlenecks identified, related factors, potential solutions, actions carried out, and next steps to be taken
3. Action and BSI	– Actions that can be taken based on the findings– Documentation of efforts to apply the solutions, including additional complications or bottlenecks encountered in the process
4. Literature review and BSI	– Search, examination, and curation of existing knowledge (global knowledge and experience) to start taking action on the bottlenecks identified and prioritized– Filling in of the BSI with this knowledge
5. Action and BSI	– Actions that can be taken based on the findings– Documentation of efforts to apply the solutions, including additional complications or bottlenecks encountered in the process
6. IR study and BSI	– Design and implementation of IR studies to further understand bottlenecks and/or potential solutions, especially in the user system– Filling in of the BSI with this new knowledge
7. Action and BSI	– Actions that can be taken based on the findings– Documentation of efforts to apply the solutions, including additional complications or bottlenecks encountered in the process

^1^BNA, bottleneck assessment; BSI, bottleneck and solution inventory; IR, implementation research.

The IS system operational model presumes that a program or intervention has already been identified and there is interest in the country, or in an implementing organization, to assess and improve its implementation. If there is a desire to begin with a broad mapping of bottlenecks, as was the case in this initiative, the process would begin with Step 1. If prior work and stakeholder consensus have already identified critical bottlenecks, the process might begin with Step 4, to identify potential solutions from the literature. If candidate solutions have already been identified, the process might begin with Step 6 to design and conduct formative research, feasibility and acceptance assessments, costing studies, effectiveness trials, or other forms of IR.

Some considerations on each of the 3 major phases and elements of the IS system operational model are provided here. For heuristic reasons this section presumes an IS initiative is beginning with Step 1 but, as noted, there are situations in which the process could begin at another point in the cycle.

#### Bottleneck assessment and BSI (steps 1–3)

In principle, a number of methods could be used to assess bottlenecks, such as highly structured surveys linked to administrative data at different levels of a delivery system (48, 49), rapid assessments within a smaller number of units within the system (50), key informant interviews, and participatory workshops (51). These vary widely in terms of the time, resources, and technical expertise involved, as well as the robustness of the findings and additional benefits each may bring. An assessment being conducted at a national level in a large country with extensive administrative and cultural diversity would require different methods than a smaller country with less diversity. PAG workshops were used in this initiative for the reasons described earlier. Regardless of the methods chosen, there is a need to prioritize bottlenecks for further attention with a transparent and systematic process. For instance, the Uganda team deployed a participatory method based on pair-ranking and conducted additional validation meetings with stakeholders not at the workshop. There is a large family of more formal, multicriteria methods available (52).

The IS system operational model shows a direct link to Actions in the center of the diagram, to remind users that many bottlenecks can be addressed through strengthened decisions, management practices, or enforcement of existing policies. Examples of such bottlenecks might be infrequent supervisory visits, inconsistent or incomplete monthly reports on service delivery, and sporadic recordkeeping for supplies management. Such problems often can be resolved by bringing them to the attention of appropriate decision makers and ensuring they are addressed, without the need for subsequent steps in the model. As shown in the figure, this would be one of the responsibilities of the knowledge broker and/or others in the delivery system who are in a position to follow up. The BSI is a complementary tool to track efforts and progress in implementing the needed changes. This is an important tool because there may be many bottlenecks and implementing their solutions may still face complications and delays.

#### Literature review and BSI (steps 4–5)

This phase of the model is another practical approach to identify solutions to those bottlenecks that are unlikely to be resolved only by tapping into CKE and strengthening existing policies and management practices. In these cases, tapping into GKE through a highly focused review of scientific and gray literature may reveal promising strategies that can be adapted to the local context. One example is to identify an adherence partner to overcome forgetfulness, a frequent bottleneck for IFAS adherence (53). Other examples might be strategies to maintain the motivation of community health workers, best practices in using mobile phones for supportive supervision, and ways to engage opinion leaders in mass media behavior change communications. Our experiences conducting practical literature reviews for topics such as this are described in a companion guide (54). Here again, there are critical roles of knowledge brokering to facilitate the reviews, engage policy and program actors in the process, and help translate the findings into actions. At this stage, the BSI is again an important tool to track progress in implementing the agreed-upon actions, recognizing that this may entail a range of complications and delays. Although literature reviews are emphasized here, these may be complemented by contacting individuals or organizations at national, regional, or global levels with recognized experience in various areas.

#### IR and BSI (steps 6–7)

This phase of the IS system operational model is presented third in sequence to emphasize that the other 2 phases should be used to the greatest extent possible, for reasons of efficiency, practicality, and timeliness. However, in some situations various forms of IR may be needed as complements during the earlier phases as well. For instance, a PAG workshop to identify bottlenecks in the delivery system may need to be complemented by a focused ethnographic study (46) or other inquiries to ensure that bottlenecks in the user system also are identified. Experience in this initiative reveals the need for a careful and systematic process to identify when and for what purpose IR is needed, as well as the most practical and appropriate methods to be used. This requires a close collaboration and negotiation between implementers and researchers, recognizing that the decision to undertake any form of IR will require ethical approval which could entail significant staff effort and delays, and diminish the time and attention for promoting action based on other steps in the cycle. Knowledge brokers can play an important role in designing and facilitating this decision process to ensure that the IR is relevant, pragmatic, and timely in relation to the needs of implementers, and to facilitate the translation and utilization of findings in collaboration with policy and program actors.

#### Knowledge brokering (required throughout all the steps)

Knowledge brokering plays 3 distinct roles in an IS system operational model. One role is to support a team of implementers and researchers to mobilize the relevant knowledge in various phases of the IS system operational model shown in Figure 3, with regards to a particular policy, program, intervention, or innovation. A second role is to ensure a strong linkage to Action in the center of the diagram so decisions can be made. A third role is to assist in building, expanding, and maintaining the IS system itself. **Table 5** summarizes the activities involved with these roles, more specifically in relation to the various forms of knowledge and steps of the IS system operational model. The latter 2 roles are further elaborated below.

**TABLE 5:** Summary of knowledge brokering activities in the IS System operational model^1^

Form of knowledge	Step of the IS system operational model	Knowledge brokering activities
Contextual Knowledge and Experience (CKE)	1. BNA	– Connect and maintain relationships among stakeholders– Gather actors from different levels– Gather preliminary data (assess local context)– Facilitate the BNA workshop– Build capacity around IS/IR– Generate buy-in among actors– Facilitate discussions– Help the actors to prioritize the bottlenecks to be addressed– Summarize the findings of the BNA– Share and validate the findings of the BNA
	2. BSI	– Compile the findings of the bottleneck assessing in the BSI– Support actors to use the knowledge (Before Action Review)– Assess and address barriers to using the knowledge
	3. Action and BSI	– Monitor knowledge use (After Action Review)– Evaluate the outcomes of using the knowledge– Compile the findings in the BSI
Global Knowledge and Experience (GKE)	4. Literature review and BSI	– Connect and maintain relationships among stakeholders– Coordinate interactions between stakeholders– Build capacity around literature review– Retrieve, organize, and share existing knowledge– Compile the findings of the literature review in the BSI– Help the actors to prioritize the next actions– Support actors to use the knowledge (Before Action Review)– Assess barriers to using the knowledge
	5. Action and BSI	– Monitor knowledge use (After Action Review)– Evaluate the outcomes of using the knowledge (After Action Review)– Compile the findings in the BSI
Contextual Implementation Research (CIR)	6. IR study and BSI	– Connect and maintain relationships among stakeholders– Facilitate negotiations and decisions about IR purposes and topics– Support actors to use the knowledge for IR (tailoring of the research questions, strengthening of a data collection tool, development of an intervention, adaptation of a research method, etc.)– Build capacity around IR activities (data collection, research method, etc.)– Assess and address future barriers to using the knowledge (Before Action Review)
	7. Action and BSI	– Monitor knowledge use (After Action Review)– Evaluate the outcomes of using the knowledge (After Action Review)– Compile the findings in the BSI
CKE and GKE	Assist in building, expanding, and maintaining a national IS system	– Form and support an IS network or initiate other strategies to foster interest, broader use, and sustainability of IS in the country – Form and maintain relationships with opinion leaders and strategic allies in academia, research institutes, nongovernmental organizations, donors, and government to act as advocates for strengthening the capacities for and practices of IS in the country– Keep abreast of developments in the science and practice of implementation by participating in virtual and in-person venues at national, regional, and/or global levels and forming strategic allies

^1^BNA, bottleneck assessment; BSI, bottleneck and solution inventory; CIR, contextual implementation research; CKE, contextual knowledge and experience; GKE, global knowledge and experience; IR, implementation research; IS, implementation science.

Establishing strong linkages to action at the center of the IS system operational model is clearly central to its effectiveness. The form that this takes varies, depending upon the scale of the program under consideration (e.g., national compared with subnational); which individuals, organizations, or structures have the authority to approve various actions; and the available opportunities to form, strengthen, or utilize interpersonal relationships between members of the country IS team and those decision makers. For instance, the strategies for linking knowledge to action in a district-based NGO project will be different from those needed for a national program managed by the nutrition unit in the Ministry of Health. Moreover, in many programs it is possible for staff at lower levels to make certain decisions, whereas staff at higher levels must be engaged to make other decisions. The knowledge broker plays a critical role in clarifying the decision makers in each case and mobilizing strategies to ensure appropriate actions are taken in response to the knowledge generated by the IS system.

The national core teams component and the knowledge brokering component were a strategy in this initiative to establish linkages and relationships among the researchers, the implementing NGOs, and senior staff in the Ministry of Health nutrition unit, but it was difficult for them to meet and become sufficiently engaged. This is often the case. However, the knowledge brokering component of an IS system is a flexible and powerful one that can be adapted to a wide variety of situations. This is because it emphasizes the importance of strategically creating, strengthening, and utilizing interpersonal relationships in order to link IS knowledge to action, even if this requires involving intermediaries who have greater access to decision makers. Those relationships can be important within formal structures established for that purpose, as was attempted with the national core teams, but they also can play a role outside of formal structures if the structures themselves do not exist, cannot be convened with sufficient frequency, or are not being responsive for any number of reasons.

The third role of knowledge brokers, assisting in expanding and maintaining the practice of IS in the country, goes beyond the responsibilities associated with the other 2 roles and is a critical step in building national capacity for IS. As shown in the final row of Table 5, it involves the exercise of strategic leadership to foster broader interest in IS (e.g., through an IS network), explore possibilities for an institutional home, forming strategic allies and relationships to support the effort, and continuing to build their own capacity for IS.

## Discussion

The present initiative was designed to generate empirical evidence on efforts to operationalize an ISN framework in the context of LMICs. The experience makes several contributions to the rapidly expanding literature on IS.

### A broader definition of IS

As an emerging science, the IS literature has considerable diversity in definitions, purposes, and frameworks (2, 10, 55). The ISN framework and the IS system operational model that emerged from this work are distinctive in several ways. They emphasize that IR is only 1 form of knowledge within the broader field of IS, highlight that many implementation bottlenecks can be addressed in a more practical and timely way by mobilizing CKE and GKE, and focus attention on knowledge utilization in addition to knowledge generation. These features are important because most of the guidance and training on how to conduct IS are, in fact, guidance on how to do or support discrete, time-bound IR projects (23, 37, 56–60).

### Guidance on operationalizing IS

This initiative generated a rich body of knowledge on the process of operationalizing the ISN framework. This includes a detailed understanding of the challenges associated with operationalizing each of the 6 IS system components and associated strategies for addressing them (Table 3); an IS system operational model for how to mobilize each of the 3 forms of implementation knowledge and link it to action (Table 4, Figure 3); and compelling evidence for the importance of knowledge brokering as an essential component of IS (Table 5). The present initiative demonstrates the need for further research on these aspects in a wider range of settings and illustrates that action research (e.g., developmental evaluation among others) is a powerful methodology for the research as well as the operationalization process itself.

### Building IS capacities

The many challenges identified during the operationalization of each of the IS system components provide some direction for future efforts to build and support future IS capacities in LMICs. Some of the challenges relate to individual understanding of IS and the skills and the mindsets needed for implementing any of the IS system components. Others relate to prevailing organizational conditions, practices, decision routines, and incentives. The large number and diverse nature of these challenges underscore the need for a system-wide approach for IS capacity building, in line with recent guidance (56). Several overall strategies proved useful in this initiative and could be included in future work. One strategy was the use of “light-touch” technical assistance and knowledge brokering, provided by 1 experienced specialist from SISN. The SISN specialist acted as a largely remote knowledge broker, coach, and mentor to the country teams. This arrangement was resource efficient (i.e., eliminated the need for more expensive full-time technical assistance in-country), fostered local ownership and accountability, and ensured that capacity-building of the local teams remained at the center of the approach. A related second strategy was the mobilization or creation of practical tools and guidance in an on-demand fashion [e.g., related to bottleneck assessments, literature reviews, IR, and knowledge brokering (47, 54)]. A third strategy was the use of developmental evaluation to provide feedback and assistance to the country teams while also documenting the forms of tools, guidance, and assistance needed in future work. Developmental evaluation is fundamentally an emergent learning approach that is increasingly recognized within the philanthropic community as a necessary strategy for working within complex systems (45).

### The way forward

Investments to build national capacity have been neglected in virtually every aspect of international development for decades, in part because of the time horizons involved and in part because of donor preferences for shorter-term project cycles, yet it is recognized that these short-term approaches are not able to produce the desired outcomes (45). In addition, there is now a recognized need to reform long-standing assumptions and practices in global health as part of an overdue decolonization agenda (61, 62). The 3 strategies used in this initiative (light-touch external assistance, practical tools and guidance, and an emergent learning approach) can be powerful and resource-efficient first steps as part of that agenda.

## Data Availability

Data described in the article, code book, and analytic code will not be made available because of the large amount of data collected during a 3-y period via multiple methods (desk review, calls, participant observation, and key informant meetings) and of the difficulty to ensure the confidentiality of a small number of key participants working at the national level in 2 countries.

## Notes

The initiative was carried out as part of a partnership with the International Initiative for Impact Evaluation (3ie) with support from Bill & Melinda Gates Foundation grant OPP1179635.

Author disclosures: The authors report no conflicts of interest.

Abbreviations used: ANC, antenatal care; BSI, bottleneck and solution inventory; CIR, contextual implementation research; CKE, contextual knowledge and experience; GKE, global knowledge and experience; IFAS, iron and folic acid supplementation; IR, implementation research; IS, implementation science; ISI, Implementation Science Initiative; ISN, Implementation Science in Nutrition; KEMRI, Kenya Medical Research Institute; LMIC, low- and middle-income country; NGO, nongovernmental organization; PAG, Program Assessment Guide; SISN, Society for Implementation Science in Nutrition; URC, University Research Co., LLC.
